# Bilateral Urinary Stones in a Transgender Woman: A Case Report in Lebanon

**DOI:** 10.7759/cureus.47958

**Published:** 2023-10-30

**Authors:** Morad Salah, Yehya Tlaiss, George Maroun, Aziz M Najjar, Imad Ghantous

**Affiliations:** 1 Urology, Saint George University Hospital in Beirut, Beirut, LBN; 2 Urology, University of Balamand, Beirut, LBN

**Keywords:** prostate, gender affirming surgery, bilateral kidney stones, transgender females, renal colic

## Abstract

This case report presents a 38-year-old transgender woman, assigned male at birth, who presented with severe right flank pain associated with nausea and hematuria. After previously undergoing gender-affirming surgeries, including abdominoplasty, liposuction, breast augmentation, and reconstructive vaginal surgery, the patient developed bilateral ureteral and kidney stones leading to significant hydronephrosis. Bilateral double J insertion was performed following a computed tomography (CT) Uroscan. This was followed by flexible ureteroscopy and laser fragmentation of the stone bilaterally. The patient's anatomy was remarkable for the presence of neovagina and prostate. This case highlights the unique challenges and considerations in managing genitourinary complications in transgender individuals. The literature is limited in the Middle East concerning transgender individuals, hence the need to conduct further research and compile comprehensive case reports on transgender individuals in the Middle East in order to establish a robust database and draw meaningful epidemiological conclusions.

## Introduction

Transgender individuals, those whose gender identity differs from the sex assigned at birth, often pursue gender-affirming medical interventions, including hormone therapy and various surgical procedures [1]. These interventions can lead to complex anatomical considerations, such as the presence of neovagina and, as highlighted in this case, the presence of a prostate. Urinary stone disease is a prevalent condition that affects both men and women with a higher likelihood in men than in women [2]. More studies are needed, however, on the transgender population to be able to gauge the prevalence in this population. Nevertheless, the current consensus considers the gender assigned from birth as both kidneys and ureters were not surgically altered in the transitioning process [3]. Additionally, it is worth noting that several factors contribute to the formation of urinary stones including warmer climates and sun exposure which lead to dehydration, diet, obesity and genetic factors as there is a 2.5 times chance of developing a ureter stone if a patient has a family history of stone disease [4]. In terms of diagnosis, non-contrast computed tomography (CT Uroscan) is the imaging method of choice due to its high specificity and sensitivity [4]. In the case of symptomatic obstruction and possible sepsis, patients should have urgent relief through either percutaneous nephrostomy, retrograde stent ureteroscopy or extracorporeal shock-wave lithotripsy [4].

Management of gender dysphoria and healthcare for transgender patients is challenging in places like the Middle East and Arab region, where transgender individuals often face significant stigmatization and discrimination, making it challenging to access healthcare services and contributing to the scarcity of case reports involving this population [5]. This case highlights the importance of addressing the unique healthcare needs of transgender individuals while recognizing and managing the possible anatomical complexities that may arise.

## Case presentation

A 38-year-old transgender woman, initially assigned male at birth, presented to the emergency room in Lebanon with severe right flank pain of one-week duration, which had worsened in the past few hours. The pain was associated with nausea and hematuria. Vital signs were within normal limits, blood pressure was 130/70 mmHg, pulse was 78 bpm, temperature was 36.7 °C, and saturation was 96% on room air. Notably, the patient had started a self-administered antibiotic course for four days without improvement. Physical examination was notable for right costovertebral angle (CVA) tenderness and a scar of an old surgery (abdominoplasty).

**Figure 1:**
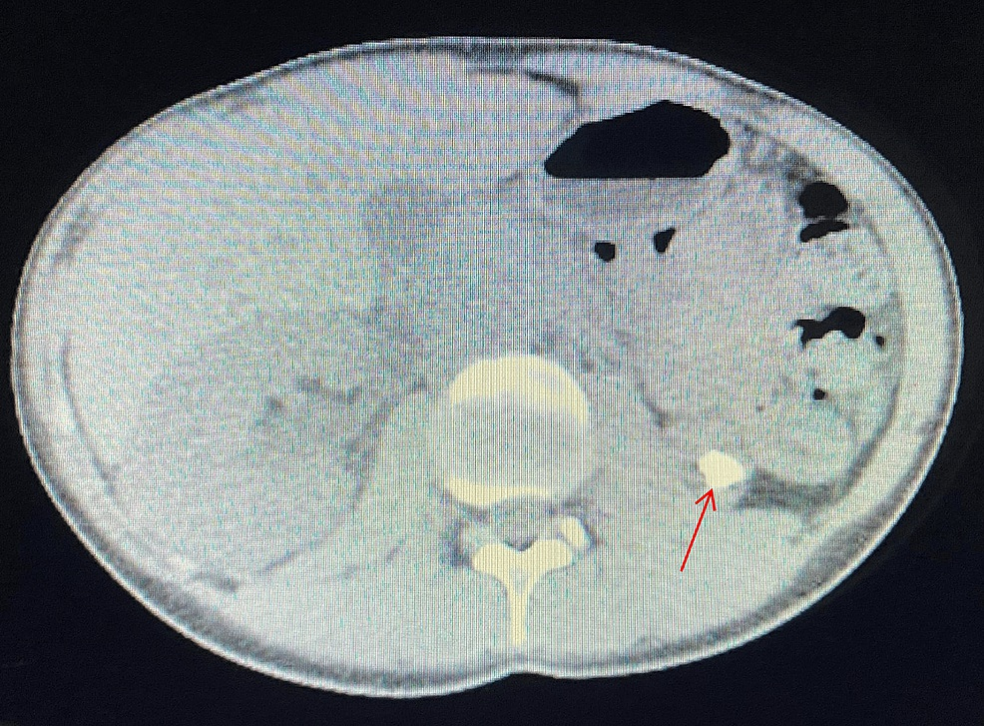
CT showing urinary stone in lower calyx of the left kidney

**Figure 2:**
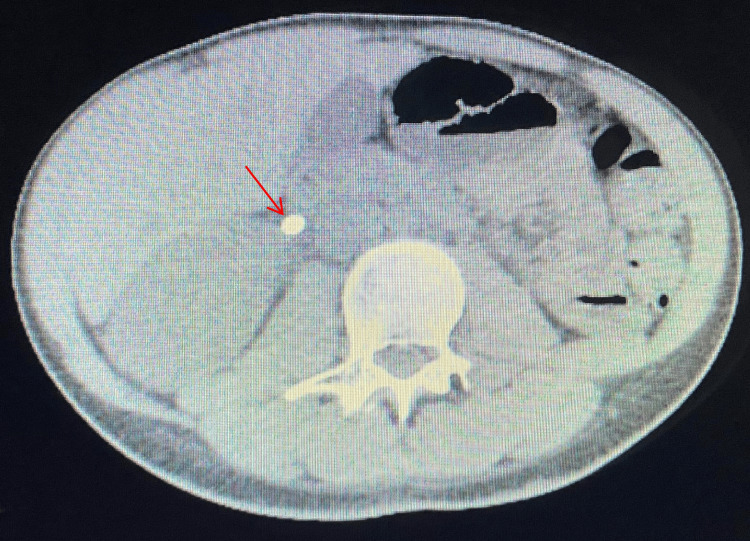
CT showing urinary stone in the right proximal ureter

The patient had a history of gender-affirming surgeries, including abdominoplasty, liposuction, breast augmentation, and reconstructive vaginal surgery, which may have contributed to her current presentation, possibly due to chronic dehydration. These surgeries altered her anatomical features, including the presence of a neovagina, which connected to a reconstructed urethra with an extended length typically found in individuals assigned male at birth. Imaging studies, including an urgent CT Uroscan, revealed bilateral kidney stones. One stone was located in the lower calyx of the left kidney that was 1.5 cm in size (Figure 1), while the right kidney stone was found in the proximal ureter and was 1.1 cm in size (Figure 2), which caused severe hydronephrosis. Given the patient's severe hydronephrosis and symptomatic kidney stones, urgent intervention was warranted. Bilateral double J insertion was performed to alleviate the symptoms and facilitate urinary drainage. This was followed one week later by flexible ureteroscopy and laser fragmentation of the stone bilaterally to remove the obstruction. One week later bilateral double J was removed. Patient symptoms including hematuria completely resolved. Interestingly, the patient's history of gender transitioning surgery six months prior added complexity to the procedure due to the altered anatomical features.

## Discussion

Transgender individuals may undergo various gender-affirming surgeries to align their physical appearance with their gender identity. These surgeries can introduce unique anatomical variations that clinicians should be aware of when assessing genitourinary symptoms. Gender-affirming therapies, including hormone therapy and surgery, can profoundly impact an individual's anatomical structure. For transgender women, hormone therapy typically involves the administration of estrogen and anti-androgens, resulting in changes such as breast development and fat redistribution. Reconstructive surgeries may include chest augmentation, facial feminization surgery, and genital reconstruction surgery [6]. These surgical interventions can introduce unique anatomical variations that healthcare providers need to consider when assessing symptoms or complications.

In the case presented here, the patient had undergone multiple gender-affirming surgeries, including abdominoplasty, liposuction, breast augmentation, and reconstructive vaginal surgery. These procedures resulted in the creation of a neovagina and a reconstructed urethra with an extended length typically found in individuals assigned male at birth. Additionally, very little research exists about the effect of gender-affirming surgeries in increasing the formation of urinary stones, mainly due to the dehydration caused by these consecutive surgeries. During long-lasting major surgeries, fluid loss occurs in different ways [7]. Hence, it is important to provide appropriate hydration after each surgery and control the intake of fluids [7]. Chronic dehydration was highlighted as a key factor for kidney stone disease in a study by Gamage et al. [8]. The process by which this occurs was described by the presence of low fluid which leads to reduced diuresis, resulting in concentrated urine which may lead to supersaturation of minerals contributing to the formation of kidney stones [8]. One study by Fascelli et al. highlighted the lower urinary tract symptoms that arise after gender-affirming surgeries and focused on transgender men rather than women [9]. The reason for this gender difference may be due presence of the prostate surrounding the upper part of the urethra, which is subject to enlargement with age and the subsequent blockage or narrowing of the urine path. These complications included incontinence, strictures, fistulae, dribbling and vaginal remnants [9]. Pereira-Lourenço et al. conducted an additional study that emphasized the significance of addressing urinary stone management post gender-affirming surgery, shedding light on lower urinary tract symptoms [10]. This research specifically documented the efficacy of employing holmium laser in the treatment of neourethral stones. Both aforementioned studies are limited by the small sample size which indicates the need for more case reports and intensive reviews. Our case report showcases a gap in the literature when it comes to urinary stones in transgender populations, especially in Lebanon and Middle Eastern countries where such cases are extremely scarce.

## Conclusions

In conclusion, this case emphasizes the importance of recognizing and addressing the unique healthcare needs of transgender people, especially in places like the Middle East where they often face challenges when seeking medical help. Gender-affirming surgeries can change a person's anatomy, making it crucial for healthcare providers to be aware of these changes when treating them for specific health issues like kidney stones, as seen in this case. Additionally, the lack of research on kidney stones in transgender populations, particularly in Middle Eastern countries like Lebanon, shows the need for more studies to understand how these surgeries might affect the risk of kidney stones. Finally, this case highlights the evolving landscape of gender-affirming interventions and their potential impact on overall health and well-being.
